# A case-controlled validation study of a blood-based seven-gene biomarker panel for colorectal cancer in Malaysia

**DOI:** 10.1186/1756-9966-29-128

**Published:** 2010-09-16

**Authors:** Kok-Thye Yip, Prashanta K Das, David Suria, Chun-Ren Lim, Guey-Hooi Ng, Choong-Chin Liew

**Affiliations:** 1 Hospital Lam Wah Ee, 141 Jalan Tan Sri Teh Ewe Lim, 11600 Jelutong, Penang Malaysia; 2 GeneNews (M) Sdn Bhd, Mount Miriam Cancer Hospital, 23 Jalan Bulan, 11200 Tanjong Bungah, Penang, Malaysia; 3 Research Department, GeneNews Ltd, 2 East Beaver Creek Road, Building 2, Richmond Hill, Ontario, L4B 2N3, Canada; 4 Department of Hematology, Brigham and Women's Hospital, Harvard Medical School, Karp Research Building, Sixth Floor, 75 Francis Street, Boston, MA, 02115, USA

## Abstract

**Background:**

Colorectal cancer (CRC) screening is key to CRC prevention and mortality reduction, but patient compliance with CRC screening is low. We previously reported a blood-based test for CRC that utilizes a seven-gene panel of biomarkers. The test is currently utilized clinically in North America for CRC risk stratification in the average-risk North American population in order to improve screening compliance and to enhance clinical decision making.

**Methods:**

In this study, conducted in Malaysia, we evaluated the seven-gene biomarker panel validated in a North American population using blood samples collected from local patients. The panel employs quantitative RT-PCR (qRT-PCR) to analyze gene expression of the seven biomarkers (ANXA3, CLEC4D, TNFAIP6, LMNB1, PRRG4, VNN1 and IL2RB) that are differentially expressed in CRC patients as compared with controls. Blood samples from 210 patients (99 CRC and 111 controls) were collected, and total blood RNA was isolated and subjected to quantitative RT-PCR and data analysis.

**Results:**

The logistic regression analysis of seven-gene panel has an area under the curve (AUC) of 0.76 (95% confidence interval: 0.70 to 0.82), 77% specificity, 61% sensitivity and 70% accuracy, comparable to the data obtained from the North American investigation of the same biomarker panel.

**Conclusions:**

Our results independently confirm the results of the study conducted in North America and demonstrate the ability of the seven biomarker panel to discriminate CRC from controls in blood samples drawn from a Malaysian population.

## Background

Colorectal cancer (CRC) is the second most common cause of cancer mortality among men and women worldwide, with an incidence of approximately 1 million cases per year and more than 500,000 deaths [1]. Although long considered a "western disease", CRC in Asia has been increasing to North American and European levels. In Malaysia, CRC is the second most common cancer in women and has recently overtaken lung cancer to become the most common cancer in men [2].

Population screening to reduce mortality from CRC has been long and vigorously advocated. However screening uptake remains less than optimal, with screening rates in North America lower than 25% to 50% [3-5]. Low compliance has been explained in part on the uncomfortable and inconvenient nature of current CRC screening tests, which, depending on the test, may require fecal samples, years of commitment, bowel preparation, time off work and may give rise to additional health risks.

We recently published a study, based in a North American population, describing a blood-based, noninvasive risk stratification tool aimed at enhancing compliance and increasing the effectiveness of current CRC screening regimens. In that study we applied blood RNA profiling and quantitative real-time RT-PCR to measure the expression of seven biomarker genes for CRC. We described a logistic regression algorithm which calculates a patient's rank, relative to the average risk population, in order to predict the patient's current risk of having CRC [6].

The biomarker panel described in that study had a sensitivity of 72% and a specificity of 70%, and was not proposed as a stand-alone test or screening tool. Rather, the panel provides information that was used to develop a risk stratification test for CRC that a clinician can use to triage patients for invasive and scarce technologies such as colonoscopy. An editorial accompanying the report describes the work as a "conceptually novel approach" that is "potentially a substantial step ahead in cancer screening technologies" [7].

In this report we tested this seven-gene biomarker panel in a Malaysian population. The Malaysian population differs from the North American in two important respects. First, the Malaysian population comprises different ethnic groups, each with different susceptibilities to CRC: Chinese Malaysians have the highest incidence rates of CRC, with an Age Standardized Rate (ASR) of 21.4 per 100,000; Indian Malaysians have an ASR of 11.3 per 100,000; and ethnic Malays have the lowest ASR of 9.5 per 100,000 [2]. Furthermore, CRC in Asian populations are more likely to be flat or depressed (non-polypoid) cancers or to arise de novo [8]. This presentation differs from western populations in which most colorectal cancers arise from precursor adenomatous polyps, which may take 10-12 years to progress to malignant cancer [9]. The specific differences in incidence between Asian groups and in the localization and distinct type of precursor lesions in the Asian populations suggest genetic variables [8].

Thus in our current study, our objective is to validate in a genetically and racially diverse Malaysian population our North American findings that a seven gene biomarker panel can differentiate colorectal cancer from controls.

## Methods

### Patient Samples

Blood samples were taken from patients referred to colonoscopy clinics in Lam Wah Ee Hospital, Penang, Malaysia, over a two-year period from August 2007 to November 2009. Patients meeting defined inclusion and exclusion criteria for the study were enrolled. Patients provided a blood sample prior to endoscopy, and anonymous clinical data was collected from each subject. Informed consent was obtained as approved by the institutions' Research Ethics Board and Joint Ethics Committee. All subjects were 21 years or older, and subjects with known, blood-borne infectious diseases (e.g. HIV, HCV) were excluded.

### Isolation of Whole Blood RNA

All blood specimens were collected prior to colonoscopy using PAXgene™tubes (PreAnalytix) and processed according to the PAXgene Blood RNA Kit protocol. Blood specimens for RNA isolation and downstream testing were kept refrigerated after collection and during transportation to GeneNews (Malaysia) Laboratory, a Standards Malaysia ISO-17025 accredited laboratory at Mount Miriam Cancer Hospital in Penang. RNA quality was assessed using Agilent 2100 Bioanalyzer RNA 6000 Nano Kit (Agilent Technologies). RNA quantity was determined by absorbance at 260 nm in a DU800 Spectrophotometer (Beckman-Coulter). The acceptance criteria for the RNA samples are: RIN ≥ 7.0; rRNA ratio ≥ 1.0 and a validated Agilent Bioanalyzer scan.

### Quantitative Reverse-Transcriptase Polymerase Chain Reaction

Quantitative reverse-transcriptase real-time RT-PCR reaction procedures for the seven gene biomarkers (ANXA3, CLEC4D, TNFAIP6, LMNB1, PRRG4, VNN1 and the duplex partner or reference gene, IL2RB) have been described previously [6]. Briefly, one microgram of RNA was reverse-transcribed into single-stranded complementary DNA (cDNA) using the High Capacity cDNA Reverse Transcription Kit (Applied Biosystems) in 1X RT reaction. For qPCR, 20 ng cDNA was mixed with QuantiTect^® ^Probe PCR Master Mix (Qiagen) and Taqman^® ^dual-labeled probe and primers corresponding to the gene-of-interest and reference gene, IL2RB, in a 25 μL reaction volume. PCR amplification was performed using a 7500 Real-Time PCR System (Applied Biosystems).

Up to 4 samples - each sample run in duplicate - can be analyzed on a single plate. Water was added to the outer wells to ensure proper temperature equilibrium. No-template controls (NTC) containing water and master mix were added to column 12 to check for possible reagent or test contamination. Column 2 and column 11 were designated for pooled blood RNA (PBR) samples for monitoring the performance of both RT and qPCR steps. PBR was prepared from blood RNA isolated from specimens collected from volunteers. Wells from row 2 to row 7 were designated for the corresponding six biomarkers, ANXA3, CLEC4D, TNFAIP6, LMNB1, PRRG4 and VNN1. IL2RB served as the reference gene for the six biomarkers.

## Results

Over the two-year period 2007 to 2009, we collected 421 blood samples, of which about one quarter were obtained from CRC patients. More than 95% of the samples passed quality control criteria (Table 1).

**Table 1:** Blood Specimen Collection and RNA Sample Quality Statistics

Collection Date	RNA QC	CASES	
		Control	CRC
August 2007	PASS	301	107
To	FAIL	8	5
November 2009	Subtotal	309	112
	Total	421	
	Passing Rate	96.9%	

CRC samples were matched to an approximately equal number of control samples for gender and age, and a total of 210 samples (99 samples from CRC and 111 from controls) were selected for this investigation. The age and sex distributions of the samples are shown in Table 2. The median age for CRC patients and controls ranged from 61 to 66. More than 80% of the samples selected were from patients more than 50 years old. The samples also reflected the multi-ethnic nature of the Malaysian population, a racial and ethnic mix quite different from the North American samples used in training and test sets (Table 3). Approximately three quarters of North American samples were from white patients; about the same percentage of the Malaysian samples were from Chinese subjects and the remainder were obtained from East Indians, Indonesians and Malays.

**Table 2:** Gender and Age Distribution in the Study Samples

Age		≤ 30	31 - 50	51 - 70	71 - 90	≥ 91	Total	Median Age
Control	Male	0	7	37	12	0	56	64
	Female	1	14	31	9	0	55	61
CRC	Male	0	7	26	18	0	51	66
	Female	1	11	22	12	2	48	62
Total Sample No.		2	39	116	51	2	210	

**Table 3:** Racial/Ethnic Composition of North American and Malaysian Samples

Patient Race/Ethnicity	North American				Malaysian	
	Training Set		Test Set			
	Control	CRC	Control	CRC	Control	CRC
Number	120	112	208	202	111	99
White	101 (84.2)	91 (81.3)	162 (77.9)	138 (68.3)	1 (0.9)	
Black	7 (5.8)	7 (6.2)	8 (3.9)	8 (4.0)		
Asian	9 (7.5)	6 (5.4)	32 (15.4)	35 (17.3)		
Chinese					74 (66.7)	70 (70.7)
Indian, East					2 (1.8)	3 (3.0)
Indonesian					32 (28.8)	21 (21.2)
Malay					2 (1.8)	5 (5.1)
Others	3 (2.5)	8 (7.1)	6 (2.8)	5 (2.5)		
Not Available				16 (7.9)		

**Note: **Percentages within individual cohort are shown in brackets.

**Figure 1:**
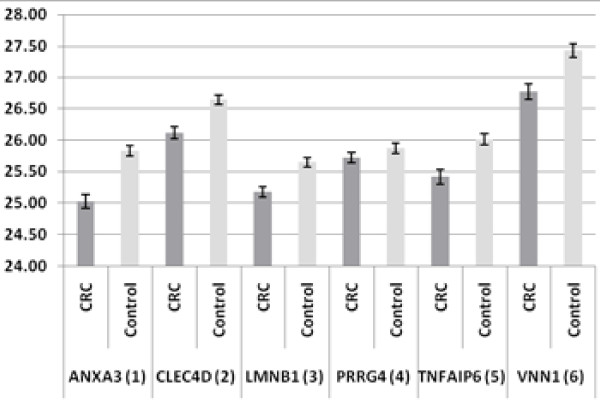
**Comparison of the Expression of Six Genes of Interest (ANXA2, CLEC4D, LMNB1, PRRG4, TNFAIP6 and VNN1) in CRC (N = 99) and Controls (N = 101) as shown in Raw Ct-values**. (Error bars denote Standard Errors of the Mean) All six biomarkers are shown as up-regulated genes in CRC as compared with controls.

**Figure 2:**
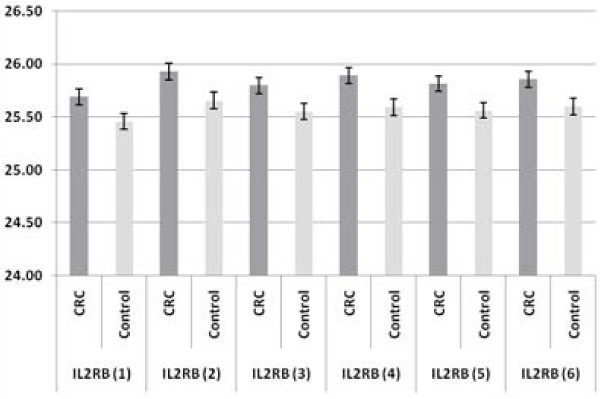
**Comparison of the Expression of Partner or Reference Gene (IL2RB) for the corresponding six biomarkers (numbered from 1 to 6) in CRC (N = 99) and Controls (N = 101)**. The figure shows the reference gene as down-regulated as compared with control samples.

Quantitative RT-PCR was performed on all the selected samples, following the protocol established in Canada [6]. Differential gene expression between CRC and control groups was estimated using the "comparative cycle threshold (ΔCt) method" of relative quantification, which normalizes the Ct values relative to the reference gene [10]. The expression of the seven-gene panel in CRC and controls is shown in Figure 1 and Figure 2. The results are shown as the average Ct for the six genes of interest (ANXA3, CLEC4D, LMNB1, PRRG4, TNFAIP6 and VNN1; numbered from 1 to 6) and their partner or reference gene, IL2RB. The error bars show the standard errors of the mean, reflecting the gene expression distributions for the seven biomarkers in the CRC and control samples. All six genes of interest are up-regulated and the reference gene is down-regulated in CRC as compared with control samples. These results confirm our finding of differential gene expression in the seven-gene panel for CRC. The relative fold-changes (CRC versus controls) for the 6 biomarkers in the Malaysian samples were compared with the data obtained from North America samples. The degree and significance of the fold changes are shown in Table 4. All six biomarkers were significantly up-regulated in CRC as compared with the control samples. The data were also evaluated using Mann-Whitney independent sample rank sum tests, and the results were highly statistically significant in both the North American and Malaysian studies (p < 0.0005).

**Table 4:** Expression of Gene Biomarkers in North American and Malaysian Samples

Symbol	Parameter	ANXA3	CLEC4D	LMNB1	PRRG4	TNFAIP6	VNN1
North	Fold Change	1.71	1.50	1.37	1.72	1.58	1.53
American	p-Value	< 0.0001	< 0.0001	< 0.0001	< 0.0001	< 0.0001	< 0.0001
Malaysian	Fold Change	2.06	1.75	1.65	1.37	1.80	1.87
	p-Value	< 0.0001	< 0.0001	< 0.0001	< 0.0003	< 0.0001	< 0.0001

**Note: **North American Training Set comprises 112 CRC and 120 control samples.

Malaysian Study Set comprises 99 CRC and 111 control samples.

The significance of the fold changes were evaluated using Mann-Whitney independent sample rank sum tests.

The performance characteristics of the Malaysian samples were demonstrated by logistic regression multivariate analysis. For the comparison study with the data obtained in North America, a common classification table cutoff or threshold value was set (P = 0.5) for the logistic regression analysis. The performance characteristics yielded a specificity of 77%, a sensitivity of 61%, accuracy of 70%, and the area under the curve (AUC) of the Receiver Operating Characteristic (ROC) was 0.76 (95% Confidence Interval: 0.70 to 0.82). These results are comparable to data obtained from the North American samples and are presented in Table 5.

**Table 5:** Comparison on Logistic Regression Analyses between North American and Malaysian Samples.

Study Location	North American		Malaysian
	Training Set	Test Set	
Sample Size	232	410	210
CRC	112	202	99
Control	120	208	111
Cut-off Value	P = 0.5	P = 0.5	P = 0.5
Area under ROC Curve (95% CI)	0.80 (0.74 - 0.85)	0.80 (0.76 - 0.84)	0.76 (0.70 - 0.82)
Significant Level	P < 0.0001	P < 0.0001	P < 0.0001
Sensitivity	82%	72%	61%
Specificity	64%	70%	77%
Accuracy	73%	71%	70%

**Note: **The MedCalc software, version 11.3 (Broekstraat 52, Mariakerke, Belgium) was used for the statistical analysis. CI denotes confidence interval.

**Figure 3:**
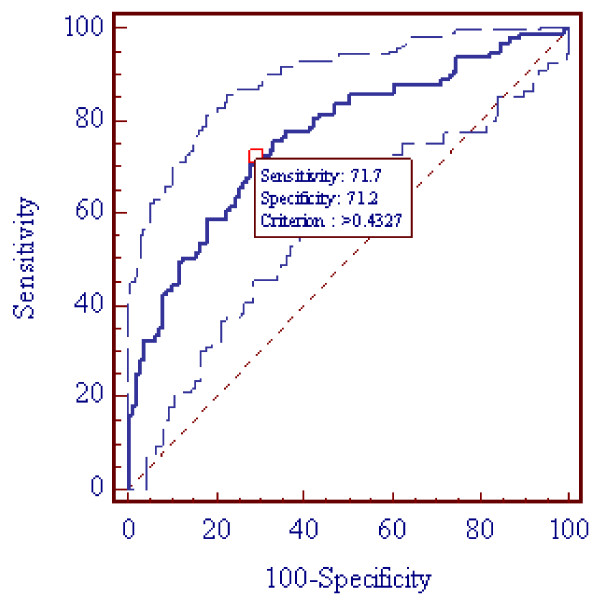
**Area Under the Curve (AUC) of the Receiver Operating Characteristic Curve (ROC) Analysis with 95% Confidence Limits (AUC = 0.76 and CI: 0.70 - 0.82) and at the Optimized Thresholds (P = 0.4327) for Sensitivity and Specificity**. **Note: **The MedCalc software, version 11.3 (Broekstraat 52, Mariakerke, Belgium) was used for the statistical analysis. CI denotes confidence interval.

The gene expression levels are continuous variables, which makes it possible to define a threshold for optimum sensitivity and specificity that is best suited for the intended application. As shown in Figure 3, at an optimized threshold (P = 0.4327) for the ROC, 71.7% sensitivity and 71.2% specificity were achieved.

**Figure 4:**
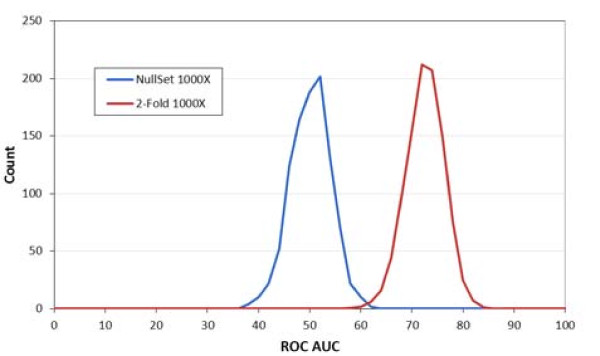
**Area Under the Curve (AUC) of the Receiver Operating Characteristic (ROC) Analysis Based on 1000X 2-Fold Cross Validation (99 CRC and 111 Control Samples)**. This chart displays the distribution for 1000 iterations of 2-Fold cross-validation using 1000 sets of randomly re-labeled samples generated from 99 CRC and 111 control samples.

The data were also subjected to 1000 iterations of 2-fold cross-validation. Figure 4 shows AUC of ROC analysis with 1000 sets of randomly re-labeled samples using data from 99 CRC and 111 controls. There is a distinct separation between the null and true data sets with only about 2% overlap; this verifies that the seven CRC biomarkers provide good power to discriminate between CRC and controls, which is unlikely due to random chance.

## Discussion

Current CRC screening programmes are complex, with multiple options. Despite efforts to establish mass population screening for CRC, screening tests remain problematic and compliance remains suboptimal [11].

Ideally, a screening procedure should be a simple and inexpensive test with a sensitivity of about 95% and a specificity about 90%. Fecal Occult Blood Tests (FOBT) are the most common tests for CRC screening, with sensitivities of about 64.3% and 81.8%, respectively for gFOBT (guaiac-based fecal occult blood test) and FIT (fecal immuno-chemical test) [12]. The effectiveness of fecal screening, however, requires patient compliance with testing over many years, and the majority of cases identified by occult blood testing are false-positives, which subjects patients to unnecessary further investigations [1]. Colonoscopy is considered the gold standard for CRC diagnosis, and is more likely to identify lesions than any other screening test. However, colonoscopy requires patient sedation, vigorous bowel preparation and carries a higher risk of complications than does other tests.

In light of the difficulties of screening, clinical practice guidelines for CRC population screening were recently updated [12], and it was concluded that "ideally, screening should be supported in a programmatic fashion that begins with risk stratification and the results from an initial test and continues through proper follow-up based on findings." Our recently introduced blood-based biomarker panel test for colorectal cancer addresses this need for risk-stratification. We showed that whole-blood gene expression profiling can stratify individuals according to their current risk of having CRC [6]. The blood-based seven-gene biomarker panel test benefits patients who wish to have information about their CRC risk status prior to considering current screening procedures. (Such patients may be uncomfortable with current screening procedures due to fear of health risks, discomfort, cultural, personal or other reasons)

The blood-based test employs receiver operator characteristic (ROC) curve analysis of the expression of six genes of interest relative to a reference gene. Continuous biomarker outputs are estimated; thus a threshold can be set to achieve a combination of sensitivity and specificity that best fits the intended use of the test. By contrast, current CRC tests such as gFOBT, FIT, fecal DNA test, are discrete, yielding yes-or-no information.

On the basis of the biomarker test, patients can be stratified by their current risk of CRC. Our calculations showed that by using our test it is possible to stratify the average risk population and select those patients with an elevated risk for CRC of 2 times or higher, such that 51% of the cancers can be found by performing colonoscopy on only 12% of the population. This is equivalent to a four-fold increase in detection rates, and can substantially increase healthcare efficiency and the use of scarce resources such as colonoscopy [6].

## Conclusion

In this study, we independently confirm that a seven-gene biomarker panel validated in a North American population is also applicable for current CRC risk stratification in a Malaysian population. The extension of the North American findings lends considerable independent validity to the blood-based CRC test, supporting the clinically utility of the risk stratification approach across different ethnicities.

## Competing interests

David Suria, Chun Ren Lim, Choong Chin Liew and Guey Hooi Ng are employees of or consultants to GeneNews Ltd, who sponsored this research.

## Authors' contributions

DS and CRL drafted the manuscript. GHN carried out the RT-PCR and data analysis; KTY and PKD examined and diagnosed the patients, collected patient records, participated in the design of the study and critically reviewed the manuscript; CCL conceived the study and critically reviewed the manuscript. All authors have read and approved the final manuscript.
